# The effects of ginger and its constituents in the prevention of metabolic syndrome: A review

**DOI:** 10.22038/IJBMS.2022.59627.13231

**Published:** 2022-06

**Authors:** Sanaz Salaramoli, Soghra Mehri, Fatemeh Yarmohammadi, Seyed Isaac Hashemy, Hossein Hosseinzadeh

**Affiliations:** 1 Student Research Committee, Mashhad University of Medical Sciences, Mashhad, Iran; 2 Department of Clinical Biochemistry, Faculty of Medicine, Mashhad University of Medical Sciences, Mashhad, Iran; 3 Pharmaceutical Research Center, Pharmaceutical Technology Institute, Mashhad University of Medical Sciences, Mashhad, Iran; 4 Department of Pharmacodynamics and Toxicology, School of Pharmacy, Mashhad University of Medical Sciences, Mashhad, Iran

**Keywords:** Diabetes, Dyslipidemia, Ginger, Hypertension, Metabolic syndrome, Obesity, Zingiber

## Abstract

Metabolic syndrome is a multifactorial disorder characterized by hyperglycemia, hyperlipidemia, obesity, and hypertension risk factors. Moreover, metabolic syndrome is the most ordinary risk factor for cardiovascular disease (CVD). Numerous chemical drugs are being synthesized to heal metabolic risk factors. Still, due to their abundant side effects, herbal medicines have a vital role in the treatment of these abnormalities. Ginger (*Zingiber officinale Roscoe*, Zingiberaceae) plant has been traditionally used in medicine to treat disorders, including CVD. The unique ginger properties are attributed to the presence of [6]-gingerol, [8]-gingerol, [10]-gingerol, and [6]-shogaol, which through different mechanisms can be beneficial in metabolic syndrome. Ginger has a beneficial role in metabolic syndrome treatment due to its hypotensive, anti‐obesity, hypoglycemic, and hypolipidemic effects. It can significantly reduce atherosclerotic lesion areas, VLDL and LDL cholesterol levels, and elevate adenosine deaminase activity in platelet and lymphocytes. Also, it promotes ATP/ADP hydrolysis. In the current article review, the critical properties of ginger and its constituents’ effects on the metabolic syndrome with a special focus on different molecular and cellular mechanisms have been discussed. This article also suggests that ginger may be introduced as a therapeutic or preventive agent against metabolic syndrome after randomized clinical trials.

## Introduction

Metabolic syndrome is a complicated disorder coming from an unhealthy diet and low physical activity (1), which is a major public-health challenge worldwide and is a notable cardiovascular risk factor (2). Besides, obesity (3), dyslipidemia (4), hyperglycemia (5), hypertension (6), and insulin resistance (7) are the most accepted unifying theories explaining the metabolic syndrome pathophysiology.

According to the National Cholesterol Education Program (NCEP) criteria, metabolic syndrome diagnosis demands at least three of the following factors to be present: central obesity or abdominal obesity: waist outline > 102 cm and > 89 cm in males and females, respectively (or body mass index (BMI) > 30 kg/m^2^), elevated plasma triglyceride (TG) ≥150 mg/dL, reduced high-density lipoprotein cholesterol (HDL) < 40 mg/dL or < 50 mg/dL in males and females, respectively; high blood pressure ≥130/85 mmHg, and fast blood sugar (FBS) of 110–125 mg/dl (8). 

Cardiovascular disease (CVD) is the most prevalent cause of human morbidity and mortality worldwide and metabolic risk factors for CVD include diabetes, high low-density lipoprotein (LDL)-cholesterol, hypertension, and obesity (9). Therefore, different efforts have been made to inhibit and cure metabolic syndrome regarding the prevention of CVD.

Herbal medicines have been used by patients with CVD around the world, for their significant preventive and therapeutic effects (10). In this regard, different studies have reported the effects of numerous plants and their active constituents in metabolic syndrome, including saffron (11), cinnamon (12), garlic (13), grapes (14), avocado (15), rosemary (16), Chinese hawthorn (17), etc. 

Ginger (*Zingiber officinale* *Roscoe*, Zingiberaceae), a medicinal plant belonging to the Zingiberaceae family, was classified by an English botanist, William Roscoe (18). Ginger with perpetual tuber or rhizome roots is cultivated naturally in Southern Asia and grows in subtropical and tropical areas (19, 20). It has been used in diet-induced metabolic disorders (19, 21, 22) and also safely in cooking and folk medicine (23). Besides, ginger is used in the treatment of arthritis (24), nonalcoholic fatty liver disease (25), primary dysmenorrhea (26), and nausea caused by pregnancy (27) and chemotherapy (28) in traditional Chinese medicine and the Indian ayurvedic system of medicine.

**Figure 1:**
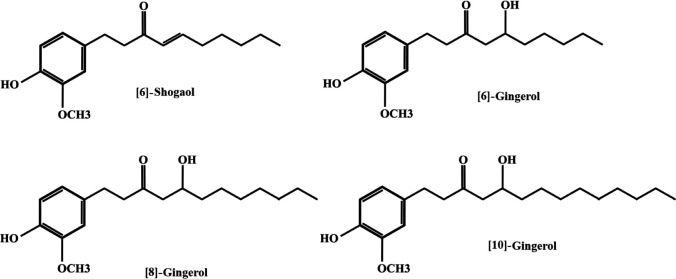
The chemical structures of ginger constituents

Rhizomes of dried ginger consist of about 6% cellulose, 8–9% fat, 9% protein, 70% carbohydrate, and 4.5% ash (20). Its main non-volatile components include [6]-gingerol, [8]-gingerol, [10]-gingerol, [6]-shogaol, which are responsible for its pharmacological activities (29, 30). The chemical structures of ginger constituents have been shown in Figure 1. Ginger and its active components are strong anti-oxidant agents (31) and have remarkable effects in the treatment of metabolic syndrome abnormalities (32, 33).

**Figure 2:**
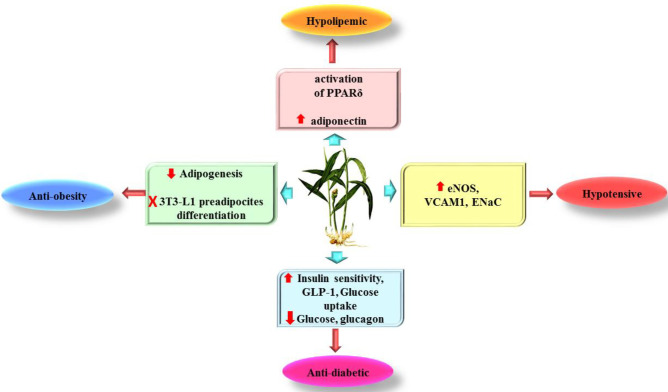
Schematic effects of ginger and its active constituents in metabolic syndrome

In the current article, the effects of ginger and its active ingredients in metabolic syndrome risk factors, including hypertension, obesity, hyperglycemia, and hyperlipidemia, were reviewed. The role of ginger and its components in metabolic syndrome have been presented in Figure 2.

## Methology

The search for the studies published was conducted in 4 databases or search engines (PubMed, Web of Science, Google Scholar, and Scopus). The literature published using the following keywords: Metabolic syndrome, Ginger; Zingiber; Diabetes; Dyslipidemia; Obesity, and Hypertension were selected and reviewed. 

**Effect on diabetes**

Numerous clinical trials have reported that metabolic syndrome is a strong predictor of diabetes incidence (34, 35). Insulin resistance is the main feature of metabolic syndrome leading to type 2 diabetes development (36). The complications attributed to diabetes, including atherosclerosis (37), retinopathy (38), nephropathy (39), and neuropathy persist as significant causes of morbidities and mortalities worldwide (40).

The hypoglycemic properties of ginger and its constituents have been mentioned in various studies. In a study, it has been shown that treatment with 200 mg/kg ethanolic ginger extract ([6]-gingerol) for ten weeks can develop insulin sensitivity in a high-fat high-carbohydrate diet-fed rat model. Thus, insulin resistance can be prevented by [6]-gingerol (41). 

In another study, the aqueous extracts of ginger were administered for six weeks (200 and 400 mg/kg- oral administration) in Sprague-Dawley rats. In this study, ginger diminished the blood glucose level and raised insulin plasma levels in obese diabetic rats (42). Similarly, the oral administration of [6]-gingerol (200 mg/kg for 28 days), induced hypoglycemia in type 2 diabetic mice, restored the interrupted endocrine factors and modulated insulin secretion in rodents (43). 

**Figure 3:**
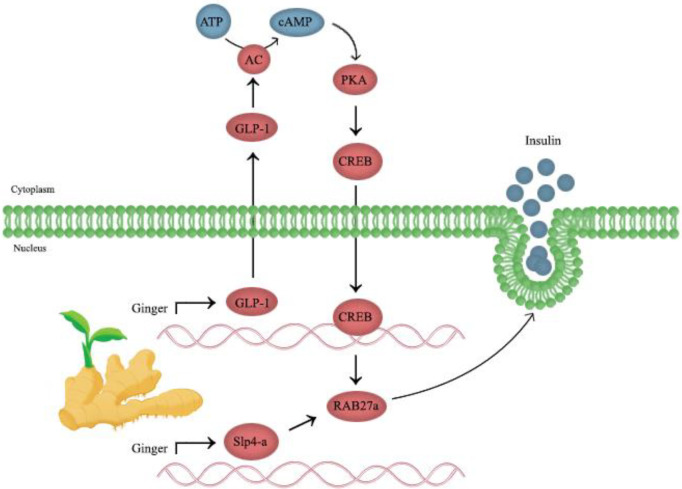
The role of ginger on insulin release

**Figure 4:**
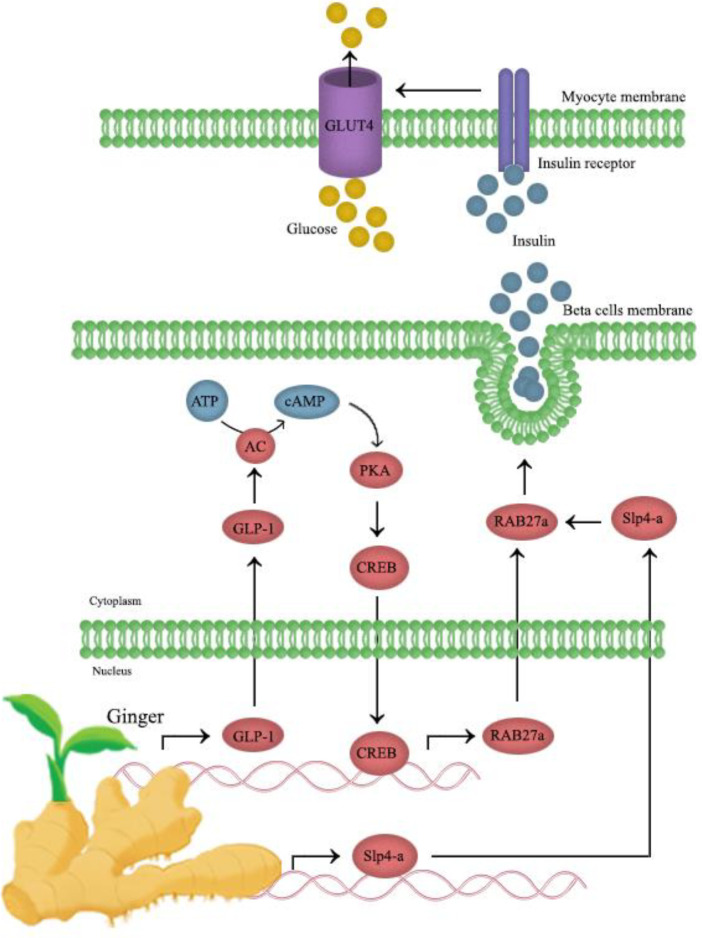
Effects of ginger on glucose consumption by skeletal myocytes

Glucagon-like peptide 1 (GLP-1) is a gut hormone released by the enteroendocrine cell that has a crucial role in stimulating insulin and suppressing glucagon release, preventing gastric depletion, and lowering appetite (44, 45).  It is investigated that GLP-1 levels can be regulated through the [6]-gingerol effect on insulin release. Mechanistically, [6]-gingerol upregulates and activates cyclic adenosine monophosphate (cAMP), protein kinase A (PKA), and cAMP response element-binding protein (CREB) in the pancreatic islets, which are major ingredients of the GLP-1-mediated insulin secretion pathway. [6]-Gingerol can upregulate both Rab27a GTPase and Slp4-a expression in pancreatic islets; Also, it improves the exocytosis of insulin-containing secretory granules. [6]-Gingerol can develop glycogen storage in muscles through arising glycogen synthase one (GYS1) activity. Moreover, there are plenty of GLUT4 transporters in the skeletal myocytes membrane, which can be explained by elevating Rab8 and Rab10 GTPases expression that is responsible for GLUT4 exocytosis to the membrane (Figure 3). Therefore, GLP-1 is regulated by the insulinotropic activity of [6]-gingerol and [6]-gingerol treatment. It promotes glucose distribution in skeletal muscles by improving GYS1 activity and boosting the presentation of GLUT4s in the cell membrane (43). The effect of ginger on glucose consumption by myocytes has been shown in Figure 4.

The effective role of [6]-gingerol in mouse skeletal muscle C2C12 cells was investigated in other research. [6]-Gingerol has a critical impact on glucose metabolism through the potentiation of insulin-mediated glucose regulation (46). 

Studies have shown that prolonged hyperglycemia can activate advanced glycation end-products (AGEs) formation and main di-carbonyl compounds levels, including methylglyoxal or glyoxal (the major originators of AGEs and N-carboxymethyl-lysine (CML)) which are significantly higher in diabetic patients (47, 48). Besides, Sampath *et al.* have investigated the administration of phloretin derived from apple and [6]-gingerol (intraperitoneal (IP) administration) for 17 weeks at two different doses (25 mg/kg and 75 mg/kg) to C57BL/6 mice on a high-fat diet reduced blood sugar, alanine aminotransferase (ALT), aspartate aminotransferase (ASP), AGEs and insulin concentrations. Also, it can reduce AGEs and CML levels via the Nrf2 (nuclear factor erythroid-2-related-factor-2) pathway, elevating the GSH/GSSG ratio, heme oxygenase-1, and glyoxalase 1 in the liver. So, phloretin and [6]-gingerol can attenuate diabetes-induced complications (49).

**Figure 5:**
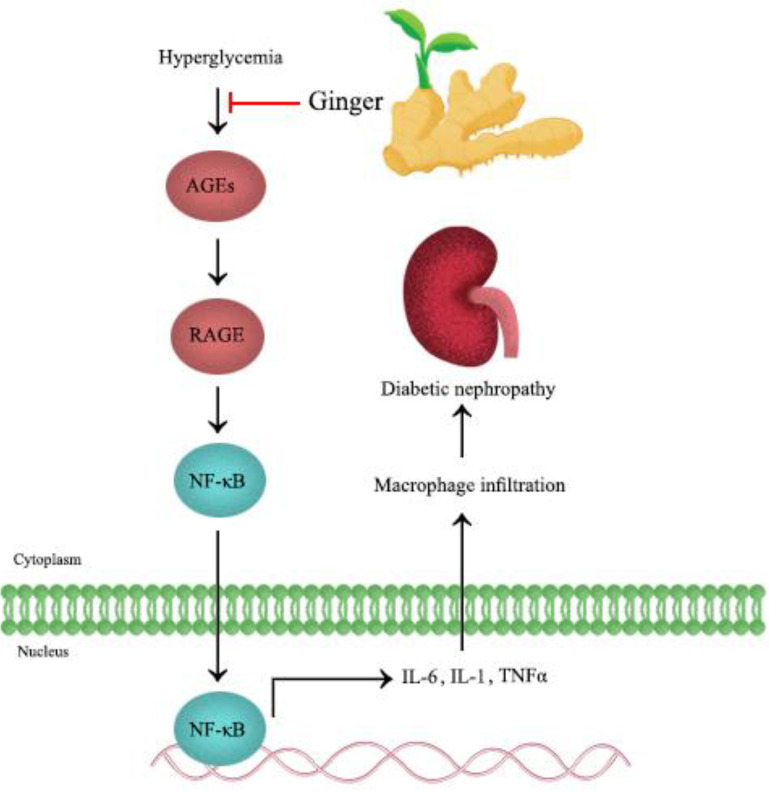
Effects of ginger on hyperglycemia

The comparison of glucose-burning effects of gingerol, shogaol, paradol, and zingerone represented that 6-shogaol and 6-paradol have substantial effects on agitating glucose consumption by 3T3-L1 adipocytes and C2C12 myotubes. Furthermore, 6-paradol, a metabolite of 6-shogaol, lowered blood glucose in the high-fat diet mouse models through oral administration of 2 different low doses (6.75 mg/kg/d) and high doses (33/75 mg/kg/d) for 30 consecutive days. Therefore, 6-paradol can be considered as an active hypoglycemic constituent of ginger (50). The effects of ginger on prolonged hyperglycemia have been presented in Figure 5.

Also, ginger may have better hypoglycemic effects in combination with cinnamon (42), garlic (*Allium sativum*) (51), and clove (52). 

It has been proven that ginger can treat type 2 diabetes complications, including nephropathy and neuropathy (53, 54). One notable mechanism involved in these complications is oxidative stress (40). So, ginger may have beneficial effects on type 2 diabetes complications due to its anti-oxidant activity. For example, in a study, rats with type 2 diabetes were treated with 400 or 800 mg/kg/d of ginger extract for six weeks (oral administration). Ginger improved hyperglycemia, hyperlipidemia, and kidney functions in diabetic animal models. Also, it reduced the histological variations in the diabetic rat’s kidney. Chronic hyperglycemia led to a considerable elevation in malondialdehyde (MDA), protein carbonyl, proinflammatory cytokines, cytochrome C, and caspase-3 levels in the rat’s kidney. Ginger extract attenuated oxidative stress, inflammation, and apoptosis; also, it boosted anti-oxidant defenses in the diabetic kidney. Thus, ginger extract is considered for a protective role against diabetic renal injuries through inhibition of oxidative stress, inflammation, and apoptosis (53). Moreover, Mata-Bermudez *et al.* study suggested that [6]-gingerol in neuropathic rats enhances antiallodynic effects which are mediated by the serotoninergic system including the 5-hydroxytryptamine receptors (5-HT_1A/1B/1D/5A _receptors) activation. These receptors mediate both excitatory and inhibitory neurotransmissions (54). Another study demonstrated that consuming ginger (5% of daily food for eight weeks) resulted in a reduction of lipid peroxidation, renal nephropathy, and elevation of plasma anti-oxidant capacity in Wistar rats (55).

On the other hand, diabetic retinopathy is a common complication of diabetes (56). Zerumbone (ZER), a compound derived from the rhizomes of *zingiber zerumbet*, was introduced as a retinal protective agent (57). Tzeng *et al.* treated STZ-diabetic rats with ZER (40 mg/kg/d- oral administration) for eight weeks; then, they claimed the administration of ZER reduces blood glucose and HbA1C. Also, ZER downregulated AGEs levels and their receptors in retinal cells of diabetic rats. Moreover, ZER attenuated the upregulation of tumor necrosis factor (TNF-α), interleukin-1 (IL-1), and interleukin-6 (IL-6) induced by diabetes. Furthermore, it ameliorated the overexpression of vascular endothelial growth factor (VEGF) and intercellular adhesion molecule-1 (ICAM-1); and reduced nuclear factor (NF)-κB activity and apoptosis in rats’ retinal cells. Thus, ZER may have a protective role in retinas due to its anti-inflammatory activity (58). 

Furthermore, the hypoglycemic and blood insulin levels increase under the influence of ginger and its active constituents have been demonstrated in a variety of clinical trial studies. In a randomized clinical trial, three-month oral administration of ginger supplementation (3 g/d) in patients with type 2 diabetes, decreased blood glucose concentration, total anti-oxidant capacity, and paraoxonase-1 (PON-1) activity (59). 

Makhdoomi Arzati *et al.* in a clinical trial study indicated that ginger supplementations (2000 mg/d for ten weeks - oral administration) could diminish FBS and hemoglobin A1C (HbA1C) concentrations in type 2 diabetic patients; but, there were no changes in the levels of TG, total cholesterol, LDL, and HDL (60). 

In this regard, another clinical trial research identified that daily receiving three 1g microcrystalline-containing capsules of ginger supplementation for eight weeks results in attenuating FBS and HbA1c levels and improving insulin resistance (61). Another clinical trial study on type 2 diabetic patients has shown that oral consumption of ginger (1600 mg/d) for 12 weeks ameliorated insulin sensitivity and reduced C-reactive protein (CRP) and prostaglandin E₂ (PGE₂). Their results demonstrated that ginger significantly decreased FBS, HbA_1C_, insulin, TG, total cholesterol, CRP, and PGE_2_ rather than in the placebo group, but there were no noticeable differences in HDL, LDL and TNFα levels. Hence, ginger might have an active role in the treatment of the complications of diabetes (62).

According to these reports, ginger has a preventive or therapeutic effect on type 2 diabetes by multiple mechanisms such as enhancing insulin levels, reducing glucose levels, increasing beta cells, inducing glucose uptakes and phosphorylation of adenosine monophosphate-activated protein kinase (AMPK), reducing insulin resistance, improving the levels of adiponectin and anti-oxidant effects. Since there is not enough evidence to improve diabetic nephropathy in clinical trials, more human studies should be conducted to validate the effects of ginger on diabetic nephropathy (40, 53, 54).

**Effect on dyslipidemia**

Patients with metabolic syndrome may have dyslipidemia, including hypertriglyceridemia, high blood levels of apolipoprotein B (apo B) and LDL, and low HDL levels (63).  It seems that the use of some plants as complementary therapeutics or extraction of their active ingredients along with currently available drugs will improve the management of hypertriglyceridemia in patients (64). Several studies have represented that ginger and its active components modified total cholesterol, total triglyceride, LDL, and HDL in serum; and have indicated that ginger is exactly a hypolipidemic agent (65-67). A study has shown that the marked elevation in total cholesterol, triglycerides (TG), lipoproteins, and phospholipids levels in serum are considerably decreased following administration of the ethanol extract of ginger (200 mg/kg for ten weeks – oral administration) in cholesterol-fed rabbits (68). 

Adiponectin directly regulates lipid metabolism. 6-Gingerol (0.2 mg/kg- 7 weeks- oral administration) improves lipid aggregation, insulin resistance, and mitochondrial dysfunction in aging rats’ skeletal muscle. 6-Gingerol decreases the high plasma TG via increasing adiponectin concentrations of plasma and adipose tissue, and it also elevates the expression of muscular adiponectin receptor 1 (AdipoR1) which activates the adenosine monophosphate-activated protein kinase/peroxisome proliferator-activated receptor-gamma coactivator-1 alpha (AMPK/PGC-1α) signaling pathway (69). Li *et al.* research has revealed that 6-gingerol by affecting lipid metabolism through increasing β-oxidation and reducing lipogenesis, normalizes the hepatic triglyceride level and plasma insulin level in the rat. Thus, the hepatic anti-steatotic effect of 6-gingerol is correlated with the upregulation of fatty acid oxidation and inhibition of the *de novo* lipogenesis pathway (69).

In a study by Hee-Jeong Kim *et al.*, ginger supplementation (200 mg/kg for 12 weeks- oral administration) reduced plasma TC and TG. It inhibited liver steatosis by regulating hepatic gene expression implicated in lipogenesis and lipolysis (70). Besides these findings, it was found that ginger (oral administration of 200 mg/Kg for seven weeks) protected alcohol-induced myocardial damage by suppressing hyperlipidemia and cardiac biomarkers in Wistar male albino rats. Ginger attenuated the alcohol-induced lipid profile changes except for HDL, so noticeably reduced the alcohol-induced myocardial damage (71).

Moreover, the role of angiotensin-1-converting enzyme (ACE) inhibitors in cardiovascular disease improvement have been established in various research studies. In a study, the inhibitory action of ACE by oral administration of two varieties of ginger (4% and 2%) for three days was investigated on high cholesterol diet-fed rats. In contrast with other studies, white ginger showed the best inhibitory effect and increased plasma lipid profile with an elevation of MDA content in rat liver and heart tissues. However, red and white ginger supplementation caused a considerable reduction in the plasma TC, TG, very low-density-lipoprotein-cholesterol (VLDL-C), LDL, and MDA levels in the tissues. Conversely, ginger supplementation increased the plasma levels of HDL. The inhibition of ACE activity may be involved in this effect of ginger (72).

Also, ginger extract (250 µg/d for ten weeks- oral administration) resulted in ameliorating plasma TG, TC, VLDL, and LDL in mice. Also, in peritoneal macrophages derived from the mice fed with ginger extract, the cholesterol biosynthesis rate was significantly reduced. Also, the ginger extract lowered the concentration of LDL-associated lipid peroxides and prevented LDL accumulation (73).

The indicators of hyperlipidemia can be cutaneous fatty acid-binding protein (C-FABP), retinoid-binding protein (RBP), and heart fatty acid-binding protein (H-FABP), which are the genes involved in lipid metabolisms. The oral administration of 500 mg ginger per day for 12 weeks tends to decrease the expression of RBP mRNA in the liver and visceral fat in hyperlipidemic rats, so, it may develop lipid metabolism in male rats (74).

It is investigated that 6-gingerol (50 to 200 μM/d) regulated hepatic cholesterol metabolism in HepG2 cells, and both cellular total cholesterol and free cholesterol (FC) were reduced. Also, 6-gingerol (100 to 200 μM) raised the LDL receptor (LDLR) protein. Moreover, it has been proven that 6-gingerol by the activation of sterol regulatory element-binding protein 2*** (***SREBP2), up-regulation of cholesterol efflux-related genes liver X receptor alpha (LXRα) and ATP-binding cassette transporter (ABCA1) can regulate cholesterol metabolism through LDLR up-regulation (75).

In line with other studies which have suggested 6-gingerol has a preventive role in adipogenesis and lipid content accumulation, another study has shown that 6-gingerol prevented the adipogenesis and attenuated mRNA transcription factors expression and the major lipogenic enzymes in the 3T3-L1 cell line. So, the role of 6-gingerol in adipogenic differentiation is associated with motivating the Wnt/β-catenin signaling activation, inducing glycogen synthase kinase-3β (GSK-3β) phosphorylation and aggregating β-catenin in nuclei (76).

Moreover, in hypothyroidism and diabetic rats that received 500 mg/kg ginger extract (oral administration) for 21 days, the level of TC and LDL significantly reduced. Also, glucose levels fundamentally decreased in ginger-treated diabetic rats (77).

Based on the studies, the use of herbal supplements may inhibit most CVDs. A 10-week intensive exercise simultaneously with ginger supplement consumption (3 g of ginger pills per day) in overweight women could ameliorate MCP-1 (type 1 monocytes chemotactic protein) without any considerable impact on ICAM-1 and interleukin 10** (**IL-10) (78). Altogether, ginger and its active ingredients reduce hyperlipidemia via multiple mechanisms (72, 73, 77), including anti-oxidant effects and increasing the level of adiponectin (69). 

**Effect on obesity**

Obesity and overweight are accompanied by various disorders, including type 2 diabetes, dyslipidemia, hypertension, and heart disease (79, 80). Obesity is a prevalent disorder worldwide for decades (81, 82). The efficacy and safety of the approved anti-obesity agents are not satisfying, so there is an essential need for new and efficient treatments (83). Based on several research studies on cell lines, animals, and humans, ginger is an anti-obesity agent (84-88).

Research by Suk *et al.* suggests that gingerenone attenuates diet-induced obesity by decreasing fat mass in mice. It suppresses the development of adipose and inflammation by AMPK activation. So, gingerenone inhibits adipogenic differentiation and lipid aggregation in the 3T3-L1 cell line (89).

The peroxisome proliferator-activated receptor δ (PPARδ) stimulators exhibited anti-obesity effects (90). A combination of 1.3% 6-shogaol and 4.8% 6-gingerol (18 weeks of oral administration) has regulatory effects on PPARδ signaling in C57BL/6J mice and is as PPARδ ligand and motivated PPARδ expression in skeletal muscle myotubes cell line. The findings represented that following the activation of the PPARδ pathway with ginger, obesity was reduced, and exercise tolerance capacity developed by elevating skeletal muscle fat catabolism (91).

Moreover, the oral administration of 5% ginger powder (4 weeks of treatment) significantly decreased body weight which was accompanied by a positive impact on the level of peroxisomal catalase without inhibition of pancreatic lipase level or any effect on bilirubin level in male albino rats (92).

In this regard, a study on male Wistar rats has shown that ginger (oral administration of 50 mg/d for 6 weeks) reduced structural heart abnormalities. They have indicated that the effects of ginger can be associated with the reduction of the leptin and cathepsin G levels via its anti-oxidant effect (93).

A clinical trial study on overweight women exhibited an insignificant effect of ginger (two 1g tablets per day for 12 weeks) on blood glucose and a notable impact on TG, rather than the placebo. Nevertheless, ginger did not show any significant impact on plasma MDA levels (94).

On the other hand, Park *et al*., in a clinical trial study, acclaimed that 6-shogaol (5.89–8.83 mg/g/d for 12 weeks- oral administration) has an anti-obesity effect without any meaningful side effects. During the supplementation period, the body weight, BMI, and body fat levels in the treatment group were significantly lower than in the placebo group (95).

Due to the different reports, the anti-obesity effect of ginger and its components is moderate. Besides, the preventive and therapeutic effects of ginger in obesity are mediated via multiple mechanisms, such as increasing leptin and HDL-cholesterol levels, elevating skeletal muscle fat catabolism, and activation of AMPK and PPARδ pathways (96-98).

**Effect on hypertension**

Hypertension is one of the main features of metabolic syndrome (6), a significant cause of CVDs, such as vascular disorders, heart disorders, and coronary artery disease (99, 100). Antihypertensive drugs have several side effects, so herbal medicines have been considered in different studies (101). Numerous studies have proven ginger, and its ingredients may have a hypotensive effect and protective impacts on the cardiovascular system (102-104).

6-Gingerol improves hypertension biomarkers expression and reduces lipid accumulation by increasing phosphorylated endothelial nitric oxide synthase (eNOS) protein, vascular cell adhesion protein 1 (VCAM1), TNF*α*, epithelial sodium channel (ENaC) protein through PPARδ in mouse preadipocytes (3T3-L1 cells), human embryonal kidney cells (HEK293 cells), and human umbilical vein endothelial cells (HUVECs) (105).

Hypertension is associated with alterations of the platelet that contributes to cardiovascular complications development. Studies demonstrated that the oral administration of 4% of ginger supplementation for two weeks elevated adenosine deaminase (ADA) activity in platelet and lymphocytes. Also, it elevated ATP/ADP hydrolysis and hydrolysis of Nω-nitro-l-arginine methyl ester hydrochloride (l-NAME) in hypertensive rats. As well, ginger increased proinflammatory cytokines (IL-1, IL- 6, interferon-γ, and TNF-α) levels with a reduction of anti-inflammatory cytokines (interleukin-10) level (106).

A research study has shown that ginger supplementations (0-2 g/d, 2-4 g/d, and 4-6 g/d- oral administration) have a preventive role on some chronic diseases, including hypertension, hyperlipidemia, type 2 diabetes, fatty liver, and CHD in men and women; also it can decrease the possibility of disorders (107). Altogether, ginger and its constituents improve blood pressure problems and related disorders. Different studies that show the role of ginger and its components on metabolic syndrome risk factors have been summarized in Table 1.

**Table 1:** Effects of ginger and its active components on metabolic syndrome risk factors

**Effect**	**Compound**	**Study design**	**Dose**	**Result**	**Ref.**
**Anti-diabetic**	6-gingerol	High cholesterol-high carbohydrate fed rats	200 mg/kg P.O.	↓Insulin resistance	(41)
	Aqueous extracts of ginger	Obese diabetic Sprague-Dawley rats	200 and 400 mg/kg P.O.	↓ BG, ↑ Insulin	(42)
	Ginger	Diabetic patients	3g/d P.O.	↓ BG	(59)
	Ginger supplementation	Diabetic patients	2000 mg/d P.O.	↓FBS, ↓ HbA1C	(60)
	Ginger powder	Diabetic patients	3 one-gr capsules P.O.	Improve insulin resistance	(61)
	Ginger supplementation	Diabetic patients	1600 mg/d P.O.	↑ Insulin sensitivity, ↑CRP	(62)
	Ginger	Diabetic Wistar rats	5% P.O.	↓ Diabetic nephropathy	(55)
	Rhizome of Ginger	High-fat diet-fed rats	100, 200 and 400 mg/kg P.O.	↑ Insulin	(108)
	Ginger methanolic extracts	Diabetic dyslipidemic rats	300mg P.O.	↓FBS	(109)
	Ginger	High-fat diet-induced type 2 diabetic rabbits	12.5% P.O.	↑ Insulin, ↓leptin	(110)
	Ginger + unripe plantain	STZ- induced Diabetic Rats	710:100 g/kg P.O.	Not effective	(111)
	Juice of Ginger methanolic extract	STZ- induced Diabetic Rats	4 mL/kg PO.	↓ BG	(112)
	Ginger extract	Diabetic Rats	500mg/kg P.O.	↓ BG	(113)
	Ethanolic ginger extract	Mice and rats	50-800 mg/kg I.P.	↓ BG	(114)
	[6]-Gingerol	As Intoxicated mice	50 mg/kg P.O.	↓ BG	(115)
	Aqueous extracts of raw ginger	Alloxan-induced diabetic and insulin-resistant diabetic rats	500mg/mLP.O.	↓ BG	(116)
	Ginger capsule	Diabetic patients	1 g/d P.O.	↓MDA	(117)
	Ginger	L6 myotubes	400 µg/mL	↓ BG	(118)
**Antilipidemic**	Ethanol ginger extract	Cholesterol-fed rabbits	200 mg/kg P.O.	↓Hyperlipidemia	(68)
	6-gingerol	Ageing rats	0.2 mg/ kg P.O.	↓ TG, ↑Adiponectin	(69)
	Ginger supplementation	C57BL/6J mice	200 mg/kg PO.	↓ TG, ↓TC	(70)
	Ginger	Male wistar rats	200 mg/Kg P.O.	↓Hyperlipidemia	(71)
	Red and White Ginger	High cholesterol diet-fed rats	4% or 2% P.O.	↓TC, ↓TG,↓MDA	(72)
	Ginger extract	Mice	25 or 250 µg/d P.O.	↓Oxidize LDL,	(73)
	Ginger	Male rat	500 mg P.O.	↑Lipid metabolism	(74)
	Ginger extract	Hypothyroidism rats	500 mg/kg P.O.	↓TC, ↓LDL	(77)
	6-gingerol	Poloxamer induced hyperlipidemic rats	3 mg/ kg I.P.	↓Hyperlipidemia	(119)
	aqueous ginger extract	Alloxan monohydrate-induced diabetic rats	1000 mg/kg P.O.	↓Hyperlipidemia, ↓TC, ↓LDL	(120)
	Gingerenone A	3T3-L1 cell line	40 μmol	↓Adipogenesis lipid accumulation	(89)
**Anti-obesity**	6-Shogaol, 6-gingerol	C57BL/6J mice	0.3% P.O.	↓Obesity	(91)
	Ginger powder	Male albino rats	5% P.O.	↓Bodyweight	(92)
	Ginger	Male wistar rats	50 mg/d P.O.	↓Leptin, ↓Cathepsin	(93)
	High-hydrostatic pressure ginger extract	High-fat diet-fed rats	8 g/kg P.O.	↓Obesity	(121)
	Extract of Ginger	Wistar rats	Unknown	↓Bodyweight	(122)
	Ginger supplements	Obese women	2 one-g tablets/d P.O.	↓Obesity	(123)
	Extract of Ginger	High-fat diet-fed rats	0.1 mL/80 g body weight P.O.	↑Muscle mitochondrial biogenesis, ↑ HDL-C, ↓Obesity	(124)
	Ethanol extract of black ginger	Diabetic NSY Mice	100 mg/kg PO.	Prevent obesity	(125)
	Ginger aqueous extract	Obese diabetic rats	200 and 400 mg/kg P.O.	↓Obesity	(42)
	Ginger aqueous extract	Obese diabetic rats	100 and 200 mg/kg P.O.	↓Obesity	(126)
**Hypotensive**	6-gingerol	3T3-L1 cells/ HEK293 cells	50 μmol/d	↓ VCAM1, ↓ TNFα, Hypertension improvement	(105)
	Ginger	Hypertensive rats	4% P.O.	↑ ADA, ↓ l-NAME	(106)
	Ginger	Hypertensive rats	4% P.O.	↑Proinflammatory cytokines, ↑ ADA, ↓ Anti-inflammatory cytokines	(106)
	Ginger	Anesthetized rats	0.3-3 mg/kg I.V.	↓ Arterial blood pressure	(127)
	Ginger aqueous extract	Anesthetized rats	3.0-10.0 mg/kg I.V.	↓ Arterial blood pressure	(128)
	Red and White Ginger	High cholesterol diet-fed rats	2-4% P.O.	Hypertension improvement	(72)
	Ginger	Patient with hypertension	0-6 g/d P.O.	↓Probability hypertension	(107)

Kg: kilogram; ml: milliliter; mg: milligram; PO: Per os (oral administration); IV: Intravenous; BG: blood glucose; FBS: fast blood sugar, HbA1C: hemoglobin A1c; CRP: c-reactive protein; TG: triglyceride; TC: total cholesterol; LDL: low-density lipoprotein; HDL: high-density lipoprotein

## Conclusion

Generally, ginger and its constituents are effective agents in the treatment of metabolic syndrome by reducing lipid accumulation by increasing the level of eNOS protein, VCAM1, TNF*α*, and ENaC. Also, ginger can play preventive or therapeutic roles in metabolic syndrome by diminishing FBS, and HbA1C by reducing insulin resistance, and anti-oxidant effects. It decreases some lipid profiles, blood pressure, and adiponectin and also increases leptin, and HDL-cholesterol levels, and elevates skeletal muscle fat catabolism as well, by activating the AMPK signaling pathway.

## Authors’ Contributions

SS Searched the literature, wrote the original draft, and revised the manuscript. FY Designed the Figures. SIH Supervised. HH and SM Designed the study and revised the manuscript.

## Conflicts of Interest

The authors declare not to have any conflicts of interest.
